# Interventions to address antimicrobial resistance: an ethical analysis of key tensions and how they apply in low- income and middle-income countries

**DOI:** 10.1136/bmjgh-2023-012874

**Published:** 2024-04-03

**Authors:** Sunil Pokharel, Bipin Adhikari, Tess Johnson, Phaik Yeong Cheah

**Affiliations:** 1 Nuffield Department of Medicine, University of Oxford, Oxford, UK; 2 Mahidol Oxford Tropical Medicine Research Unit, Faculty of Tropical Medicine, Mahidol University Phayathai Campus, Bangkok, Thailand; 3 Ethox Centre, Nuffield Department of Population Health, University of Oxford, Oxford, UK

**Keywords:** Public Health, Infections, diseases, disorders, injuries

## Abstract

Antimicrobial resistance (AMR) is a global health and one health problem. Efforts to mitigate the problem of AMR are challenging to implement due to unresolved ethical tensions. We present an in-depth ethical analysis of tensions that might hinder efforts to address AMR. First, there is a tension between access and excess in the current population: addressing lack of access requires facilitating use of antimicrobials for some populations, while addressing excessive use for other populations. Second, there is a tension between personal interests and a wider, shared interest in curbing AMR. These personal interests can be viewed from the perspective of individuals seeking care and healthcare providers whose livelihoods depend on using or selling antimicrobials and who profit from the sales and use of antimicrobials. Third, there is a tension between the interests of current populations and the interests of future generations. Last, there is a tension between addressing immediate health threats such as pandemics, and AMR as a ‘silent’, chronic threat. For each of these tensions, we apply ‘descriptive ethics’ methods that draw from existing evidence and our experiences living and working in low-income and middle-income countries to highlight how these ethical tensions apply in such settings.

**Summary box**

- Antimicrobial resistance (AMR) is a super-wicked one health problem.
- We identify ethical tensions that need to be considered in designing context-specific interventions to mitigate AMR, especially in low-income and middle-income countries.
- AMR policies need to balance the interests of all relevant stakeholders. Our actions against AMR need to consider the interests and well-being of future generations. We also need to ensure that the chronic and ‘silent’ threat of AMR receives adequate attention and allocation of resources as we continue to deal with the acute and ‘loud’ threats of public health emergencies.

## Introduction

Antimicrobial resistance (AMR) is a serious concern that threatens human, environmental and animal health.^1 2^ AMR is a complex ‘one health’ problem, that is, a problem where we must acknowledge that the health of people is interconnected to the health of animals and the health of environment to address the problem properly. Recent estimates suggest that in 2019, nearly 5 million deaths were associated with bacterial AMR, including 1.3 million deaths directly attributable to AMR.^3^ In addition, the burden of AMR falls disproportionately on low-income and middle-income countries (LMICs) and on the most vulnerable in societies, such as children under 5 years, those who lack access to healthcare, and those living in the margins of societies, such as migrant and refugee populations. The burden of resistance will increase substantially if bacterial infections, which continue to be the major contributors of the mortality in children below the age of 5 years globally, become resistant.^4^ The same goes for malaria infections, which, in 2021, resulted in 619 000 deaths with the majority in children under 5 years, and would again transfer to a burden attributable to AMR if these infections became resistant to antimalarials.^5^ The populations vulnerable to AMR are the same populations vulnerable to climate change—the poor and most disadvantaged in societies. They are often least responsible for the impacts of AMR and climate change and will probably be the most burdened by interventions to curb AMR and climate change.

Given the seriousness of the problem, AMR may initially seem like a cut-and-dried issue, with a clear moral imperative to reduce use of antimicrobials. However, AMR is an issue that still requires further ethical analysis, because of the complex, unresolved ethical tensions it raises. These tensions may result partly from the nature of AMR itself, involving inherent conflicts, and partly from inadequacies and incoherence in the development of some interventions proposed to address AMR.

First, consider the nature of AMR, which has been considered a ‘super-wicked’ problem.^6^ ‘Super-wicked’ problems are difficult to solve in part due to their interrelatedness with other problems, and because they do not respond to standard problem-solving mechanisms.^7^ They are also difficult to solve because of their urgency, because those seeking the problem are sometimes part of the cause, because those authorities to address the problem are either weak or non-existent and because policy responses to these problems tend to discount the future irrationally.^6^

Second, consider problems with potential solutions to mitigate the spread of AMR. AMR occurs naturally, and is thus to some extent inevitable, but much of the problem of AMR is due to human behaviours. Inaction against AMR will potentially lead to inability to treat even minor infections or perform minor surgeries in the future. Although urgent actions are necessary, the potential interventions against AMR are intricately complex and could also exacerbate existing inequalities. Conflicting courses of action in various parts of the world and context-specific need for antimicrobials complicates potential solutions to the problem.

Third, due to the multisectoral impacts associated with AMR, a ‘one health’ approach is necessary. Concerted efforts from various disciplines are needed to provide solutions for human, animal and environmental health. However, there are challenges in balancing the competing interests of multiple economic and political sectors and organisations involved in animal, human and environmental health.

Many solutions have been proposed to mitigate AMR such as outlawing sales on over-the-counter (OTC) antimicrobials,^8^ taxing use of antibiotics for self-limiting illness,^9^ incentivising pharmaceutical industries to accelerate innovation rather than just tweaking existing drugs,^10^ withdrawing sales-based incentives by pharmaceutical companies^11^ and using the concept of the ‘duty of easy rescue’ to appeal to healthy individuals to forgo antimicrobials for mild and self-limiting infections.^12^ However, these solutions have not taken into consideration ethical tensions that are inherent in AMR or how these tensions play out in specific contexts, particularly LMICs. Many scholars have published on ethical issues related to AMR but not specifically those related to interventions to mitigate AMR, and the existing literature on ethical analysis lacks analysis using an LMIC lens.

National action plans (NAPs) on AMR, stemming from the WHO global action plans, are now available for at least 108 countries, including LMICs. However, the implementation of NAPs has been challenging due to the inherent ethical tensions that we will address in this paper. Also, the role of patients, the public and communities in AMR solutions is often overlooked in designing and implementing NAPs and resolving these ethical tensions.

Our analysis focuses on the ethical tensions that need to be taken into consideration when designing any policies and interventions related to AMR, particularly to address the problem as it presents in some LMIC contexts. Using and building on experiences of the problem of AMR and potential solutions from specific contexts to design appropriate policy for that context is essential. Ethical tensions refer to conflicts or dilemmas that arise when different ethical principles, values or considerations come into conflict, making it challenging to make decisions or take actions that fully align with all of these principles. These tensions often arise in situations where there is no straightforward answer or clear solution that can address all ethical concerns simultaneously.

We provide an ethical analysis of the tensions that stakeholders in AMR interventions may face, first by synthesising existing normative literature and applying ‘normative ethics’ methods to more thoroughly characterise theoretical tensions faced by stakeholders. Second, we apply ‘descriptive ethics’ methods to highlight whether and how these theoretical ethical tensions apply in the LMIC setting, particularly in countries with fewer resources.^13^ In our normative ethical analysis, we identify four distinct ethical tensions that we ought to take into account when designing any interventions to address AMR. For each of these tensions, we draw from evidence and experience living and working in LMICs to further assess whether and how these ethical tensions apply in LMIC settings, specifically.

In the global health literature, epistemic power often still lies primarily with researchers in high-income countries (HICs). The knowledge generated through HICs creates a body of literature that may be well adapted to the ethical tensions and concerns in some HIC contexts, and possibly some LMIC contexts. However, it is a matter of epistemic justice as well as the development of ethically appropriate and effective AMR policy to ensure more literature on AMR is built on learning from researchers based in or with experience working or living in LMIC settings. Our initial normative analysis is primarily guided by the existing literature, with our original contribution being the analysis of whether these often-generalised or universalised issues identified in existing normative literature are, indeed, generalisable to LMIC settings. To do so, we draw on some of our own experiences living in LMICs, interacting with communities (eg, via community advisory boards, community clinics, clinical trials and primary healthcare services in remote communities)^14^ and conducting research on the issues related to AMR in LMICs, particularly South and Southeast Asia and Sub-Saharan Africa.^14–20^ The ethical tensions laid out in this article are thereby juxtaposed with day-to-day lived experiences of stakeholders in AMR interventions in LMICs, particularly the practicalities of health services and patients’ interactions with both formal and informal healthcare providers. Aligning with our experiences, we focus more on human health and less on animal and environmental health.

## Key tensions for interventions to address AMR and how they apply in LMICs

In this section, we undertake a normative analysis to characterise four key ethical tensions that need further consideration in policy and intervention design. For each of these tensions, we then apply ‘descriptive ethics’ methods which highlight whether and how these ethical tensions apply in LMICs in particular where there are fewer resources. We recognise that some of these tensions are not confined to LMICs. Some of these tensions are also prevalent in HIC settings.

### Tension between excess and access

It is viewed that the use of antimicrobials is the single, most important factor leading to AMR, even when medically indicated and used appropriately.^21^ In many parts of the world, there is an excess of antimicrobials and overuse of them in human, agricultural and environmental sectors, while in other parts of the world there is lack of access. Within countries and subpopulations, in particular in LMICs, the tension between excessive use and lack of access may also exist, for example, between the private sector, where there may be excess, and the public healthcare sector, where there may be lack of access.^22^ In contexts of impoverished communities, with substantial discrepancies between public and private healthcare systems and healthcare dependent on out-of-pocket expenses, individuals’ financial capacities to buy expensive private healthcare often determines access to antimicrobials. Therefore, the experiences of this tension in LMICs are particularly acute, even compared with the theoretical, global-level characterisation of the ethical tension. Affluent individuals or those with fewer constraints regarding healthcare choices in LMICs might have the opportunity to obtain the latest and pricier broad spectrum antimicrobials, including last resort options that are typically inaccessible, while the poor might struggle to access essential medicines or first-line antimicrobials.

It is often challenging to delineate, even within a community, who has excess and who has lack of access and the reasons behind it. For example, Thailand has Universal Health Coverage and the cost of antimicrobials is low due to widely available generics as well as drugs produced by the Thai Government Pharmaceutical Organisation, hence for most people antimicrobials are cheap and easily available. However, some people still purchase drugs from informal stores due to, for example, cost, distance from the nearest official clinic and long waiting times. Hence, there is a tension between making antimicrobials more available in some countries, regions or communities, while limiting the use of antimicrobials in others. While reducing excess and increasing access may require opposite interventions, both must be balanced, and targeted appropriately, to address AMR effectively.

In places with poorly resourced health systems, a lack of or delay in access to antimicrobials causes more deaths than deaths due to drug-resistant infections. Yet, lived experiences of the access side of the tension have been little discussed in previous normative theoretical analyses. In some countries with poorly resourced health systems, lack of access to both off-patent and new drugs is in fact a greater problem than their excessive use.^23^ In many low-resource settings, there is a lack of access to healthcare including lack of adequate clinical expertise where it is needed, the lack of laboratory facilities to identify the causative agent and lack of good quality antimicrobials.^24^ For instance, in remote mountainous villages of Nepal, female community health volunteers without adequate medical training are often the only front-line workers. While people have to travel long distance to access health facilities, a shortage of qualified health workers and a fragmented supply chain for essential medicines has placed the population at risk of deaths due to diarrhoea, respiratory and febrile illnesses and several other curable infections.

In Sub-Saharan Africa, prereferral rectal artesunate has been shown effective at reducing mortality in cases of severe malaria by several studies, particularly for those with malaria live in rural communities where transport to a clinic where they can have parenteral drug administration is limited.^25^ Initially, this may look like a case of excess, where a prereferral antimalarial treatment is given when infection is only suspected. However, looking at how the tension is experienced by people living rurally in African countries who do not have fast access to other treatment, the tension loses some of its prior theoretical normative force. The key issue in this case is not excessive use, but the issue of lack of access to diagnostic tools and parenteral antimalarials in rural communities. Until access in these communities is improved, there is no feasible alternative for the parents of children with suspected severe malaria who may face death or severe disability before they can reach a clinic.

In many places, especially in LMICs, many people buy drugs OTC from informal stores and pharmacies. For example, in Nepal, the OTC use of antimicrobials constitutes a major source of antimicrobial use where antimicrobials are bought without prescription or medical supervision.^26^ The excess–access tension, then, does not only relate to prescribed uses of antibiotics, as sometimes assumed in the normative literature to date, but rather also relates to an excess of OTC uses of antimicrobials which may not be appropriate to the individual’s infection, and an issue of accessing prescribed antimicrobials that are appropriate to the individual’s infection, in some LMICs. While OTC sales of antimicrobials can promote access to antimicrobials where they are needed, they also lead to potential excessive and inappropriate use where antimicrobials are purchased for mild or self-limiting conditions. While there are health and AMR-related consequences, OTC access to antimicrobials can be a great relief to those who would have to otherwise travel long distances or cannot afford to see a qualified healthcare provider, such as in rural Nepal, Thailand and Laos.^16 27–29^ Stringent regulations against OTC use of antimicrobials in contexts with better alternative healthcare access and security can help improve care and optimise antimicrobial use, while such an approach can be counterproductive or even fatal, such as in the case of severe malaria, in remote communities where access to alternative options for healthcare is lacking.

### Tension between personal interests and shared interests

The personal interests relevant for this ethical tension can be grouped into four overlapping categories. These are the (1) interests of those seeking care, (2) the interests of healthcare professionals treating individuals seeking care, (3) the interests of those whose livelihoods depend on selling antimicrobials and (4) the interests of those who make large profits from the use and sales of antimicrobials. The interests of these groups of individuals may conflict with the broadly shared goal of protecting antimicrobial effectiveness.

Broadly, this tension has been illustrated in the normative ethical literature by framing AMR as a ‘tragedy of the commons’.^9^ Antimicrobial effectiveness can be considered as a common-pool resource,^9^ such as a pasture that is available to herdsmen and becomes degraded from overgrazing.^30^ It is in herdsmen’s short-term interests to allow their cattle to graze on the pasture, even if they know this will lead to the pasture being depleted. This analogy is not completely accurate because, for example, the increase in individual consumption does not necessarily result in progressively greater benefits.^31^ However, the analogy is still useful in an initial discussion of antimicrobials, demonstrating how, if individuals act out of self-interest by using, prescribing or selling antimicrobials, then everyone, including those individuals, may be harmed in the future when antimicrobials are no longer effective.

#### Interests of the individuals seeking care

As a patient, it is natural to want the ‘best’ drugs to treat one’s illness. While everyone should have an interest in preserving antimicrobial effectiveness, this regard for the future and for the wider community is only secondary or none at all in the case of communities with no awareness of AMR. This happens for both self-limiting illness as well as severe illness, and true for those in low-resource and high resource settings. In low-resource settings, antimicrobials have become a ‘quick-fix’ infrastructure for fractured health systems, as well as for care, productivity, hygiene and structural inequalities.^32^ Patients take antimicrobials when ill in the hope, rightly or wrongly, that they can go to work, be productive and continue to provide care to others. Indeed, where people do not have access to clean water or healthcare services, they may resort to taking antibiotics routinely. Highlighting lived experiences of this tension, in Malaysia, people buy drugs from the pharmacy without a prescription as it is quicker and cheaper than seeing a doctor. In Nepal, patients often self-prescribe and demand for antimicrobials for their fever or mild respiratory symptoms as it is perceived to provide them a blanket cure rather than asking for and complying with medical advice which is perceived to be more expensive and delaying care. In Laos and Thailand, combination of drug–cocktails is popular OTC options for all kinds of ailments. In Sub-Saharan Africa, prereferral rectal artesunate is life-saving for a child with fever who may have severe malaria. The tragedy of the commons is sometimes apparent in these cases, where the individual benefits at a cost to the antimicrobial commons, and sometimes not, where the increasing risk of an individual becoming the host of a resistant pathogen is great enough to outweigh the potential benefit. The conflict between individuals’ and the public’s interests is exacerbated where regulations are less strict, enforcement is lacking or corruption is rampant, as this may allow patients to demand antibiotics inappropriately (eg, to treat viral diseases or for mild and self-limiting infections) and antimicrobials to be sold illegally. If patients seeking antimicrobials are denied, they may seek alternative care or purchase antimicrobials from private hospitals, pharmacies or informal sellers.^16 24 27^

#### Interests of the healthcare provider

An important ethical tension in relation to the use of antimicrobials is the moral responsibility of the healthcare provider to serve the individual patient versus moral obligations towards broader public health goals of curbing the spread of AMR. In normative terms, this can be described as a tension between the principles of clinical ethics and those of public health ethics. Clinical ethics principles require that healthcare providers place the emphasis on the health and well-being of the individual person seeking care.^31^ Using the lens of clinical ethics, the problem of perpetuating AMR would appear to be secondary. By contrast, public health ethics prioritises improving the health of a population.^33–35^ This holds as an experienced tension, too, where in many LMIC settings, clinicians may not only straddle the public and private sectors, but individual versus group-level healthcare, and may have little information to inform decisions where they must weigh public against individual health when deciding whether to prescribe an individual an antimicrobial.

Healthcare providers often prescribe antibiotics because of the uncertainty, and the concern that the patient may have a serious bacterial infection, and in some cases to fulfil patients’ demands,^24^ and professional culture norms such as ‘defensive medicine’, where clinicians need to worry about receiving complaints or being sued.^36^ Primary care physicians in LMICs routinely face the challenges in making clinical decisions due to the lack of adequate diagnostics and clear guidelines for prescribing antimicrobials. In the lack of clinical decision support tools, judgements made for empirical antimicrobial use could have disadvantages in relation to the costs and potential side effects, but withholding antimicrobials from those needing them urgently can cost lives. Fearing legal repercussions and aiming to ensure patient safety and satisfaction, clinicians often admit to resorting to prescribing antimicrobials ‘just in case’ an infection might be present.

Clinicians in most LMICs, for instance in Sri Lanka, are employed by the public sector but also work in private hospitals due to low wages in public hospitals.^24^ The clinicians serving private healthcare are aware that patients can choose to ‘shop around’ for other clinicians until obtaining their preferred prescriptions. In India, owing to the large population and low health-worker-to-patient ratio, patients who can afford it often go to private clinics where they are offered advanced antimicrobials in the hope of expediting recovery. In these settings, clinicians offering antimicrobials can build goodwill and thus their businesses. They are incentivised by extra income from private practice and may feel pressured to prescribe broad spectrum antibiotics irrespective of their need—both to ensure ‘good’ treatment and also to maintain the clients.^24^

Healthcare workers in places with poor healthcare facilities may, furthermore, offer antimicrobials to desperate patients to replace more comprehensive care that is unavailable.^32^ These drugs serve to replace healthcare personnel, healthcare infrastructure and to correct for hygiene issues.^32^ For instance, healthcare providers routinely use antibiotics as prophylaxis for their surgical procedures because of inadequate sterilisation of patient rooms and medical tools.^32^

#### Interests of those who depend on antimicrobials for their livelihoods

People across many sectors depend on antimicrobials for their livelihoods and their businesses. The use of antibiotics in agriculture involves the administration of antibiotics to farm animals such as cattle, pigs and chickens. Especially in LMICs, human-critical classes of antibiotics are routinely used as therapies, prophylaxis, and in some cases to promote livestock growth particularly in intensive farming systems where animals are kept in close quarters and are therefore more susceptible to disease outbreaks.^37 38^ Tensions between these stakeholders’ interests and a shared interest in curbing AMR may be particularly salient in lived experiences of farmers in some LMICs.

While farmers’ interests in crop livestock health and growth may seem insignificant in comparison to a wider interest in the antimicrobial effectiveness, there may also be a wider public interest in farmers’ continued use of antimicrobials. Diseased crops or livestock mortality may threaten food security and lead to calls for intensified, antimicrobial-heavy food production. However, this comes with its own risks for food safety, as shown associations between antimicrobial use in broiler chicken farming and resistant genes.^39^

Another group of people that relies on antimicrobials for their livelihoods and businesses are the owners and employees of small pharmacies and drug outlets. Pharmacies, drug outlets and informal shops are reliant on sales of drugs to keep their business going in addition to not disappointing their patients. In some places, animal grade antibiotics are repackaged for human use. In LMICs, antimicrobials remain the key components of inpatient and outpatient medical prescriptions, and the major source of sales and revenues.^40 41^ For example, informal stores in Thailand sell unlabelled drug packets called *‘yaa chud’* that contains antimicrobials.^42^

For the farmers, owners of pharmacies and informal shops, and others who rely on antimicrobials for precarious livelihoods and businesses, curbing AMR is not a priority in comparison to making a living or sustaining their businesses.

#### Interests of those who profit from the manufacture and sales of antimicrobials

The fourth set of personal and shared interests that may conflict relates to those who make large profits from the manufacture and sales of antimicrobials (beyond just making a living or sustaining their businesses), including pharmaceutical companies, manufacturers, big corporations as well as politicians. While these interests may not initially appear to create ethical tensions, the continued revenue and production is essential to ensure the availability of the antimicrobials.

Pharmaceutical companies earn revenue through the sale of their products to healthcare providers, pharmacies, and in some cases, directly to patients. The current model of sustaining the pharmaceutical industry is based on volume of sales. In the absence of an alternative model, the profit-based incentive structure is necessary to ensure a healthy supply of therapeutics. The industry is highly competitive. The return on investments in research and development of new antimicrobials is lower than for other drugs.^43^ This is worse for antimicrobials for diseases that primarily affect the poor such as tuberculosis and malaria. Unfortunately, LMICs suffer disproportionately from these major diseases. In addition, increasing pressure on pharmaceutical companies to reduce their marketing of broad spectrum antimicrobials to reduce overselling of antimicrobials have been responded by reducing the productions.^11^ While this progress is encouraging, it is inadequate to resolve the tension between these interests.

### Tension between the interests of current populations and the interest of future generations

The third tension engenders questions about intergenerational justice,^44^ and our responsibility today for the well-being of future generations.^45^ We can characterise this, normatively, as a tension between the interests and well-being of future people in continued antimicrobial effectiveness, and the interests of current people in using antimicrobials when they are ill or fear becoming ill. Intergenerational justice includes future people among ‘all people’ who should be treated equitably. It implies that in making decisions that will affect future people, we recognise the importance of not unfairly harming their future opportunities and quality of life.^46^ From a sustainability justice lens, future people’s interests should be considered in our decision-making.

If we continue ‘socially discounting’ the effects of our decisions on future people as not equally morally important, then today’s actions and inactions could lead to great intergenerational injustice.^45^ It may not be fair to leave future generations without effective antimicrobials, yet there are no consensus on balancing the interests of future generations and their right to health in current decisions.

Bringing in the lens of lived experiences in LMICs, it is worth noting that there is an intersection between this intergenerational justice and justice in the geographical distribution of the burdens of AMR. We can expect that future generations in LMICs may bear the more burden from AMR compared with HICs, as their healthcare and political systems are less well resourced, and thus less resilient to the impacts of AMR. These impacts might include increased healthcare expenditure, loss of lives, food insecurity and economic loss. There might be even further reason, then, to prioritise the interests of these future people whose ancestors today are among the worse off, globally, and whose disadvantage on that basis may be badly exacerbated through the burdens of AMR. In our experience working in low-resource settings, where immediate concerns such as basic needs, healthcare access and political instability take precedence, current people’s concern about issues such as AMR affecting future generations is limited. In such settings, people’s focus is often on addressing more immediate and pressing issues, such as securing food, water, shelter and basic healthcare. Long-term issues that may primarily affect future people or their distant future selves, such as AMR, might not be a priority when people are struggling to meet their basic needs.

### Tension between addressing immediate public health threats and chronic threats

The final tension arising in the context of policies and interventions to address AMR is between addressing public health emergencies that might be considered acute, immediate, ‘loud’ threats to health and addressing AMR, which is thought of as a chronic, ‘silent’ or ‘hidden’ threats.^47^ AMR is a chronic threat to human and animal health and the environment. By contrast, public health emergencies can be defined as events ‘whose scale, timing or unpredictability threatens to overwhelm routine capabilities’,^48^ which might include pandemics, bioterrorism and natural disasters. These immediate threats attract attention and aid quickly.^49^ Recently, we have faced public health emergencies that have attracted much more attention, sometimes at the expense of efforts to address AMR. During the COVID-19 pandemic, in particular, resources were directed toward reducing infections and deaths from COVID-19.^50^ These actions may have come at the cost of increasing the number of future deaths from drug-resistant infections.^51^ The visibility of the impact of COVID-19 including the broadcasting of daily national statistics of cases of COVID-19 and the formation of high profile COVID-19 specific task force in many countries have perpetuated this. This level of attention contrasts with the relative lack of attention on AMR across the globe but more pronounced in LMICs where the awareness of AMR is low. Broad spectrum antibiotic sales were positively associated with COVID-19 cases globally during 2020–2022.^52^ Healthcare providers may offer empirical antibiotics to avoid uncertainty of the origin of infections and sometimes to prevent from potential secondary infections. In many LMICs such, the OTC sales of antibiotics without prescriptions soared during the early phase of the COVID-19 pandemic. Many perceived antibiotics to be effective for a wide variety of illnesses, including the respiratory symptoms consistent with COVID-19. The fear and uncertainty attached to COVID-19 and the potential stigma if and when diagnosed made patients hesitant to visit formal healthcare services and instead resorted to informal health services.

## Conclusion

We have conducted a comprehensive analysis, both characterising normative ethical tensions in efforts to address AMR and exploring these tensions through the lenses of lived experiences and how the tensions may be more or less salient in LMIC contexts, especially in places with fewer resources. We have discussed the fundamental conflicts that complicate efforts to control the proliferation of AMR and provided some illustrations from literature and our own observations. Resolving these conflicts necessitates context-specific solutions that are contingent on the prevailing political landscape, national regulations, disparities in societal structures, cultural and professional norms, the autonomy of medical practitioners in prescription practices, the perceived significance of antimicrobial agents and other relevant factors. Context-specific solutions require dialogue and in-depth engagement to include voices from the most vulnerable communities.^17^ Interventions to curb the spread of AMR and AMR NAPs should consider the ethical tensions outlined in our analysis. If well-meaning interventions fail to take these tensions into consideration, the most vulnerable communities could end up shouldering the largest burdens. Solutions to curb AMR are not straightforward and there will be trade-offs. The ‘just transitions’ framework, which gained prominence in climate governance, may be able to offer a new approach to balance these trade-offs.^53^ It could serve as a starting point for discussions prioritising justice, sustainability, inclusivity and equity. The notion of a just transition highlighted questions of equity and distribution of burdens in the climate context, as well as questions of procedural justice and how to ensure all voices are heard. Applying this approach in AMR could help ensure that any interventions to curb AMR addresses inequalities and trade-offs across sectors and societies with differing interests and ethical tensions highlighted in our paper. The populations vulnerable to AMR are the same populations vulnerable to climate change—the poor and most disadvantaged in societies, and they are often least responsible for the impacts of both AMR and climate change and will probably be the most burdened by interventions to curb AMR and climate change.

## Data Availability

There are no data in this work.

## References

[1] Viens AM , Littmann J . Is antimicrobial resistance a slowly emerging disaster? Public Health Ethics 2015;8:255–65. 10.1093/phe/phv015 26566396 PMC4638061

[2] Balasegaram M , Clift C , Røttingen J-A . The global innovation model for antibiotics needs reinvention. J Law Med Ethics 2015;43:22–6. 10.1111/jlme.12270 26243239

[3] Murray CJL , Ikuta KS , Sharara F , et al . Global burden of bacterial antimicrobial resistance in 2019: a systematic analysis. The Lancet 2022;399:629–55. 10.1016/S0140-6736(21)02724-0 PMC8841637 35065702

[4] Institute for Health Metrics and Evaluation . Global burden of disease study. Seattle, 2019. Available: https://ghdx.healthdata.org/gbd-2019

[5] World Health Organization . World malaria report. 2021. Available: https://www.who.int/teams/global-malaria-programme/reports/world-malaria-report-2022

[6] Littmann J , Viens AM , Silva DS . The super-wicked problem of antimicrobial resistance. In: Jamrozik E , Selgelid M , eds. Ethics and drug resistance: collective responsibility for global public health. Public health ethics analysis. Cham: Springer, 2020. Available: 10.1007/978-3-030-27874-8_26)

[7] Rittel HWJ , Webber MM . Webber MM dilemmas in a general theory of planning. Policy Sci 1973;4:155–69. 10.1007/BF01405730

[8] Jacobs TG , Robertson J , van den Ham HA , et al . Assessing the impact of law enforcement to reduce over-the-counter (OTC) sales of antibiotics in Low- and middle-income countries; a systematic literature review. BMC Health Serv Res 2019;19:536. 10.1186/s12913-019-4359-8 31366363 PMC6670201

[9] Giubilini A . Antibiotic resistance as a tragedy of the Commons: an ethical argument for a tax on antibiotic use in humans. Bioethics 2019;33:776–84. 10.1111/bioe.12598 31107562 PMC6767468

[10] Global AMR R&D Hub & World Health Organisation . Incentivising the development of new Antibacterial treatments. 2023. Available: https://globalamrhub.org/wp-content/uploads/2023/05/2.-G7_FULLReport_HUB_WHO_FINAL_10052023.pdf [Accessed 13 Aug 2023].

[11] Mahase E . Antibiotic resistance: more companies are withdrawing sales reps or incentives in order to limit use, finds report. BMJ 2020:m235. 10.1136/bmj.m235 31964654

[12] Singer P . Famine, affluence, and morality. Philos Public Aff 1972;1:229–43.

[13] Georgetown University Press . Methods in medical ethics. 2nd edn. 2010.

[14] Cheah PY , Lwin KM , Phaiphun L , et al . Community engagement on the Thai-Burmese border: rationale, experience and lessons learnt. Int Health 2010;2:123–9. 10.1016/j.inhe.2010.02.001 22984375 PMC3442337

[15] Pokharel S , Raut S , Adhikari B . Tackling antimicrobial resistance in low-income and middle-income countries. BMJ Glob Health 2019;4:e002104. 10.1136/bmjgh-2019-002104 PMC6861125 31799007

[16] Adhikari B , Pokharel S , Raut S , et al . Why do people purchase antibiotics over-the-counter? A qualitative study with patients, Clinicians and dispensers in central, Eastern and Western Nepal. BMJ Glob Health 2021;6:e005829. 10.1136/bmjgh-2021-005829 PMC8118002 33975888

[17] Poomchaichote T , Osterrieder A , Prapharsavat R , et al . AMR dialogues: a public engagement initiative to shape policies and solutions on antimicrobial resistance (AMR) in Thailand. Wellcome Open Res 2021;6:188. 10.12688/wellcomeopenres.17066.2 35505979 PMC9034167

[18] Cheah PY , Jatupornpimol N , Suarez-Idueta L , et al . Understanding a science-themed puppet theatre performance for public engagement in Thailand. Wellcome Open Res 2018;3:7. 10.12688/wellcomeopenres.13239.1

[19] Swe MMM , Hlaing PH , Phyo AP , et al . Evaluation of the forum theatre approach for public engagement around antibiotic use in Myanmar. PLoS One 2020;15:e0235625. 10.1371/journal.pone.0235625 32645036 PMC7347174

[20] Tindana P , Guissou R , Bolarinwa OA , et al . Ethical considerations in deploying triple artemisinin-based combination therapies for malaria: an analysis of stakeholders' perspectives in Burkina Faso and Nigeria. PLoS One 2022;17:e0273249. 10.1371/journal.pone.0273249 36083995 PMC9462557

[21] CDC (Center for Disease Control and Prevention) . About antimicrobial resistance. 2015. Available: https://www.cdc.gov/drugresistance/about.html [Accessed 7 Jan 2022].

[22] Paruk F , Richards G , Scribante J , et al . Antibiotic prescription practices and their relationship to outcome in South Africa: findings of the prevalence of infection in South African intensive care units (PISA) study. S Afr Med J 2012;102:613–6. 10.7196/samj.5833 22748439

[23] Laxminarayan R , Matsoso P , Pant S , et al . Access to effective antimicrobials: a worldwide challenge. The Lancet 2016;387:168–75. 10.1016/S0140-6736(15)00474-2 26603918

[24] Krockow EM , Tarrant C . The International dimensions of antimicrobial resistance: contextual factors shape distinct ethical challenges in South Africa, Sri Lanka and the United Kingdom. Bioethics 2019;33:756–65. 10.1111/bioe.12604 31264232 PMC6771635

[25] Gomes MF , Faiz MA , Gyapong JO , et al . Pre-referral rectal artesunate to prevent death and disability in severe malaria: a placebo-controlled trial. Lancet 2009;373:557–66. 10.1016/S0140-6736(08)61734-1 19059639 PMC2646124

[26] Morgan DJ , Okeke IN , Laxminarayan R , et al . Non-prescription antimicrobial use worldwide: a systematic review. Lancet Infect Dis 2011;11:692–701. 10.1016/S1473-3099(11)70054-8 21659004 PMC3543997

[27] Haenssgen MJ , Charoenboon N , Zanello G , et al . Antibiotic knowledge, attitudes and practices: new insights from cross-sectional rural health behaviour surveys in low-income and middle-income South-East Asia. BMJ Open 2019;9:e028224. 10.1136/bmjopen-2018-028224 PMC6707701 31434769

[28] Adhikari B , Phommasone K , Pongvongsa T , et al . Treatment-seeking behaviour for febrile illnesses and its implications for malaria control and elimination in Savannakhet province, Lao PDR (Laos): a mixed method study. BMC Health Serv Res 2019;19:252. 10.1186/s12913-019-4070-9 31018855 PMC6480816

[29] Marahatta SB , Yadav RK , Giri D , et al . Barriers in the access, diagnosis and treatment completion for tuberculosis patients in central and Western Nepal: a qualitative study among patients, community members and health care workers. PLoS One 2020;15:e0227293. 10.1371/journal.pone.0227293 31940375 PMC6961875

[30] Hardin G . The tragedy of the commons. Science 1968;162:1243–8. 10.1126/science.162.3859.1243 5699198

[31] Jamrozik E , Heriot GS . Ethics and antibiotic resistance. Br Med Bull 2022;141:4–14. 10.1093/bmb/ldab030 35136968 PMC8935610

[32] Denyer Willis L , Chandler C . Quick fix for care, productivity, hygiene and inequality: reframing the entrenched problem of antibiotic overuse. BMJ Glob Health 2019;4:e001590. 10.1136/bmjgh-2019-001590 PMC6703303 31497315

[33] Kass NE . An ethics framework for public health. Am J Public Health 2001;91:1776–82. 10.2105/ajph.91.11.1776 11684600 PMC1446875

[34] Childress JF , Faden RR , Gaare RD , et al . Public health ethics: mapping the terrain. J Law Med Ethics 2002;30:170–8. 10.1111/j.1748-720x.2002.tb00384.x 12066595

[35] Upshur REG . Principles for the justification of public health intervention. Can J Public Health 2002;93:101–3. 10.1007/BF03404547 11968179 PMC6979585

[36] Broom A , Kirby E , Gibson AF , et al . Manners, and medical ritual: defensive medicine and the fetish of antibiotics. Qual Health Res 2017;27:1994–2005. 10.1177/1049732317721478 28737082

[37] European Medicines Agency . Sales of veterinary antimicrobial agents in 31 European countries in 2019 and 2020. Luxembourg, 2021.

[38] US Food and Drug Administration . Summary report on antimicrobials sold or distributed for use in food producing animals. 2021.

[39] Luiken REC , Van Gompel L , Munk P , et al . Associations between antimicrobial use and the faecal resistome on broiler farms from nine European countries. J Antimicrob Chemother 2019;74:2596–604. 10.1093/jac/dkz235 31199864 PMC6916135

[40] Nga DTT , Chuc NTK , Hoa NP , et al . Antibiotic sales in rural and urban pharmacies in northern Vietnam: an observational study. BMC Pharmacol Toxicol 2014;15:6. 10.1186/2050-6511-15-6 24555709 PMC3946644

[41] Wang J , Wang P , Wang X , et al . Use and prescription of antibiotics in primary health care settings in China. JAMA Intern Med 2014;174:1914–20. 10.1001/jamainternmed.2014.5214 25285394

[42] Sunpuwan M , Punpuing S , Jaruruengpaisan W , et al . What is in the drug packet?: access and use of non-prescribed poly-pharmaceutical packs (Yaa Chud) in the community in Thailand. BMC Public Health 2019;19:971. 10.1186/s12889-019-7300-5 31331304 PMC6647088

[43] Shlaes DM . Antibacterial drugs: the last frontier. ACS Infect Dis 2020;6:1313–4. 10.1021/acsinfecdis.0c00057 32527094

[44] Rawls J . A theory of justice. Cambridge MA. Belknap Press of Harvard University Press, 31 December 1971. 10.4159/9780674042605

[45] Littmann J , Buyx A , Cars O . Antibiotic resistance: an ethical challenge. Int J Antimicrob Agents 2015;46:359–61. 10.1016/j.ijantimicag.2015.06.010 26242553

[46] Parfit D . Reasons and persons. Oxford University Press, 1986. 10.1093/019824908X.001.0001

[47] Mahoney AR , Safaee MM , Wuest WM , et al . The silent pandemic: emergent antibiotic resistances following the global response to SARS-Cov-2. iScience. iScience 2021;24:102304. 10.1016/j.isci.2021.102304 33748695 PMC7955580

[48] Nelson C , Lurie N , Wasserman J , et al . Conceptualizing and defining public health emergency preparedness. Am J Public Health 2007;97 Suppl 1(Suppl 1):S9–11. 10.2105/AJPH.2007.114496 17413078 PMC1854988

[49] World Health Organization . Health emergencies. 2023 Available: https://www.who.int/our-work/health-emergencies

[50] Johnson T . A trade-off: antimicrobial resistance and COVID-19. Bioethics 2021;35:947–55. 10.1111/bioe.12928 34464462 PMC8652952

[51] Getahun H , Smith I , Trivedi K , et al . Tackling antimicrobial resistance in the COVID-19 pandemic. Bull World Health Organ 2020;98:442–442A. 10.2471/BLT.20.268573 32742026 PMC7375214

[52] Nandi A , Pecetta S , Bloom DE . Global antibiotic use during the COVID-19 pandemic: analysis of pharmaceutical sales data from 71 countries, 2020-2022. EClinicalMedicine 2023;57:101848. 10.1016/j.eclinm.2023.101848 36776504 PMC9900305

[53] Varadan SR , Chandler CIR , Weed K , et al . A just transition for antimicrobial resistance: planning for an equitable and sustainable future with antimicrobial resistance. The Lancet September 2023. 10.1016/S0140-6736(23)01687-2 37696277

